# Visfatin (NAMPT) affects global gene expression in porcine anterior pituitary cells during the mid-luteal phase of the oestrous cycle

**DOI:** 10.1186/s40104-024-01054-z

**Published:** 2024-07-09

**Authors:** Kamil Dobrzyn, Grzegorz Kopij, Marta Kiezun, Ewa Zaobidna, Marlena Gudelska, Barbara Zarzecka, Lukasz Paukszto, Agnieszka Rak, Nina Smolinska, Tadeusz Kaminski

**Affiliations:** 1 Faculty of Biology and Biotechnology, University of Warmia and Mazury in Olsztyn, Olsztyn, Poland; 2 School of Medicine, Collegium Medicum, University of Warmia and Mazury in Olsztyn, Olsztyn, Poland; 3 Institute of Zoology and Biomedical Research, Jagiellonian University, Krakow, Poland

**Keywords:** Alternative splicing, Anterior pituitary, Differentially expressed genes, lncRNA, Oestrous cycle, Pig, RNAseq, Transcriptome, Visfatin

## Abstract

**Background:**

The pituitary belongs to the most important endocrine glands involved in regulating reproductive functions. The proper functioning of this gland ensures the undisturbed course of the oestrous cycle and affects the female’s reproductive potential. It is believed that visfatin, a hormone belonging to the adipokine family, may regulate reproductive functions in response to the female’s metabolic state. Herein we verified the hypothesis that suggests a modulatory effect of visfatin on the anterior pituitary transcriptome during the mid-luteal phase of the oestrous cycle.

**Results:**

RNA-seq analysis of the porcine anterior pituitary cells revealed changes in the expression of 202 genes (95 up-regulated and 107 down-regulated in the presence of visfatin, when compared to the non-treated controls), assigned to 318 gene ontology terms. We revealed changes in the frequency of alternative splicing events (235 cases), as well as long noncoding RNA expression (79 cases) in the presence of the adipokine. The identified genes were associated, among others, with reproductive system development, epithelial cell proliferation, positive regulation of cell development, gland morphogenesis and cell chemotaxis.

**Conclusions:**

The obtained results indicate a modulatory influence of visfatin on the regulation of the porcine transcriptome and, in consequence, pituitary physiology during the mid-luteal phase of the oestrous cycle.

**Supplementary Information:**

The online version contains supplementary material available at 10.1186/s40104-024-01054-z.

## Introduction

The pituitary is one of the most important endocrine organs in the organism. This small, inconspicuous structure, through the secretion of several tropic hormones, mediates the functioning of several target organs such as adrenals and gonads. Due to its structure, consisting of the nervous and glandular parts, the pituitary processes the central nervous system derived signals into the hormonal commands forwarded to the endocrine tissues. Due to its location, and regulatory abilities, the pituitary is a crucial point of the regulatory loops, such as the hypothalamic-pituitary-adrenal (HPA) or hypothalamic-pituitary-gonadal (HPG) axes. The secretory function of the anterior pituitary (AP) may be regulated not only by the hypothalamic-derived releasing hormones, like growth hormone releasing hormone or gonadoliberin, or the hormones sent as the feedback response from target tissues such as luteinizing hormone (LH), follicle-stimulating hormone (FSH) or prolactin (PRL), but also by several other factors and hormones, like steroid hormones, prostaglandins, cytokines, neuropeptides, or activin, inhibin and follistatin [1–7]. A growing body of evidence indicates that the pituitary actions may also be modulated by hormones involved in metabolism regulation such as adipokines.

Adipokines, a group of hormones produced mainly by the white adipose tissue, exert a pleiotropic action in the organism, however, their primary function is the integration of energy metabolism. Previous studies indicated the regulatory effect of adipokines, i.a., leptin, adiponectin, chemerin or apelin, on the secretory functions of the pituitary [8–16]. Visfatin (Nicotinamide phosphoribosyltransferase, NAMPT, VIS), also included in the adipokines group, was described for the first time in 1994 as a pre-B cell colony enhancing factor (PBEF) [17]. The hormone was qualified as an adipokine when its insulin-mimetic effects were proven for the first time [18]. The human *NAMPT* gene is located at 7q22.3 and encodes 52–54 kDa protein product [17, 19]. NAMPT protein may exist in the organism in two distinct isoforms: intra- (iNAMPT) and extracellular (eNAMPT) [20], from which iNAMPT is considered an intracellular enzyme and the adipokines’ specific actions are addressed only to the extracellular form. Until now no specific VIS receptor has been identified.

Besides its insulin-mimetic actions, VIS was found to exert proinflammatory influence through the up-regulation of cytokines production in monocytes [21]. It has also been proved, that adipokine acts as an anti-apoptotic and pro-angiogenic agent [22, 23]. The hormone has also been reported as a stimulatory factor in several tissues tumorigenesis (for a review see: [24]). Several metabolic disorders, such as obesity, polycystic ovary syndrome, type 2 diabetes or atherosclerosis have been correlated with VIS plasma level deviations [25–28]. NAMPT localisation in the endocrine glands, like ovaries, testes, mammary gland, pituitary, or in the hypothalamus, the structure responsible for the regulation of the above glands, indicates its potential involvement in the endocrine axes regulation [29–34].

The results of our previous studies indicating the modulatory influence of VIS on LH and FSH secretion by the porcine anterior pituitary cells (APc) [35] inspired us to set up a hypothesis assuming the regulatory role of adipokine in this important gland. Herein, we verified the hypothesis, assuming the modulatory effect of VIS on the global gene expression in the porcine APc. The studies were conducted using Illumina Next Generation Sequencing (NGS) technology. The obtained RNAseq results were used for the analysis of differentially expressed genes (DEGs), as well as for the determination of changes in the expression of long noncoding RNAs (DELs) and frequency of alternative splicing events (DASs). The revealed DEGs were assigned to specific Gene Ontology (GO) categories and analysed for the potential interaction networks. Since the secretory functions of the pituitary are subjected to the cyclic changes related to the phase of the oestrous cycle of the animals, the research was conducted with the use of animals from d 10 to 12 of the oestrous cycle, when the secretory activity of the corpus luteum (CL), and hence, whole HPG axis is high, but does not change as dynamically as in the case of the follicular phase. Moreover, on those days, the secretion of luteinizing (LH), as well as follicle stimulating (FSH) hormones does not change as dynamically as during the pre- and postovulatory phases of the cycle which could affect the obtained results [36, 37]. Another justification for choosing this particular period is a very similar activity of CL during the mid-luteal (d 10–12) phase of the cycle and during gestation, the obtained herein results may, in the future, serve for the investigation of the VIS effect on the HPG axis during pregnancy.

## Materials and methods

### Animals and tissue collection

All studies were conducted in following the Polish Act on the protection of animals used for scientific or educational purposes, enacted on January 15, 2015 (Polish Journal of Law of 2015 item 266) as well as directive 2010/63/EU of the European Parliament of September 22, 2010, on the protection of animals used for a scientific purpose. For the experiment, the glandular parts of the pituitaries were obtained from the crossbreed gilts (age of 7–8 months and weight of 130–150 kg) on d 10 to 12 of the oestrous cycle and descended for slaughter at the commercial abattoir. The day of the oestrous cycle was confirmed based on the morphology of the ovaries (large, centrally closed, pink corpora lutea, soft in touch, with the inner cavity filled with luteal tissue and blood vessels at the surface; presence of medium, 4.0–6.0 mm of diameter, follicles) [38]. Within a few minutes after slaughter, APs were placed in ice-cold phosphate buffer solution (PBS) enriched with glucose and a mix of antibiotics (Antibiotic Antimycotic Solution, Merck, USA), and immediately transported to the laboratory. The number of APc isolated from one pituitary gland was approximately 9 to 10 million, whereas about 18 million APc were necessary to conduct one experiment (*n* = 1). Therefore, 2 to 3 APs were collected and digested together. The in vitro cell cultures were conducted in five independent replicates (*n* = 5).

### In vitro cell cultures and total RNA isolation

The APc were isolated as described by Kawamata et al. [39], with modifications. The cells were washed with Dulbecco’s medium (Sigma-Aldrich, USA) enriched with 0.1% bovine serum albumin (Sigma-Aldrich, USA) and antibiotics (Antibiotic Antimycotic Solution, Merck, USA), and homogenized with a scalpel blade. The homogenates were digested with 0.2% collagenase solution (Sigma-Aldrich, USA) at 37 °C for 30 min. Isolated APc were centrifuged three times at 1,000 × *g* (10 min, room temperature) in 15-mL tubes containing Dulbecco’s medium. The remained tissue was treated with 0.2% collagenase and 0.25% pancreatin (Sigma-Aldrich, USA) for 10 min. The digestion steps were repeated until the complete digestion of the material. After each digestion, the obtained cells were collected and washed in Dulbecco’s medium, as described above. The cell suspensions were pooled and filtered through a 70-μm mesh filter. The total amount and viability of APc were estimated using a haemocytometer and trypan blue dye (Sigma-Aldrich, USA), the mean percentage of viable cells after isolation was 95.65% ± 1.3%. The cells were suspended in McCoy’s 5 A medium (Sigma-Aldrich, USA), enriched with 10% horse serum (Sigma-Aldrich, USA) and 2.5% foetal calf serum (Sigma-Aldrich, USA) and seeded onto 6-well culture plates (1.5 × 10^6^ cells/2 mL medium/well). The cells were preincubated for 72 h at 37 °C in a water-saturated atmosphere with 5% CO_2_ and 95% air. After 48 h of preincubation, 1 mL of fresh, serum-free McCoy’s 5 A medium was added to each well. After preincubation media were replaced with fresh, enriched McCoy’s 5 A medium and incubated for another 24 h in the presence of human recombinant VIS (final concentration of 100 ng/mL; Cat. # 8424-VF, RD System, USA). The hormone concentration was determined based on Reverchon et al. [32] and our preliminary studies. Since VIS was diluted directly in McCoy’s medium, cells descended for the control group (*n* = 5) were incubated in the pure medium without the presence of any treatment. The potential effect of VIS on cell viability was excluded with the use of the Alamar Blue test. Cell viability and toxicity of the treatment were determined using the alamarBlue™ assay according to the manufacturer’s instructions. The mean percentage of viable cells after incubation was 103.6% ± 6.2%.

After the incubation, total RNA from APc was isolated using Extrazol (BLIRT, Poland), following the manufacturer’s protocol. The purity (A_260_/A_280_) and quantity (wavelength 260 nm, A_260_) of the obtained RNA samples were determined spectrophotometrically using Infinite M200 Pro (Tecan, Männedorf, Switzerland). Bioanalyzer 2100 (Agilent Technology, USA) was used to determine the RNA integrity. Only samples which passed RNA integrity number (RIN) > 8 criteria were qualified for the further transcriptome high-throughput sequencing (RNA-Seq) analysis and the quantitative real-time polymerase chain reaction (qPCR) and reverse transcription polymerase chain reaction (RT-PCR) validations.

### Library construction and sequencing

The NGS sequencing procedure, as well as preliminary bioinformatic analysis, was conducted by LCSciences (USA). Approximately 5 µg of total RNA was used to remove ribosomal RNA (rRNA) from the samples with the use of Ribo-Zero Gold rRNA Removal kit (Illumina, USA). The remaining RNA samples were fragmented into short fragments using divalent cation buffers at elevated temperatures (NEBNext^®^ Magnesium RNA Fragmentation Module, NEB, USA). The obtained samples were reverse-transcribed to cDNA using SuperScript™II Reverse Transcriptase (Invitrogen, USA). Subsequently, cDNA was used to synthesise U-labeled second-stranded DNA using *E. coli* DNA polymerase I (NEB, USA), RNase H (NEB, USA) and dUTPSolution (Thermo Fisher, USA). The obtained double-stranded cDNA (dscDNA) was subjected to the end repair and A-tailing processes. In the end, the obtained fragments of dscDNA were ligated with specific adaptors. Quality control analysis and the quantification of the obtained libraries were performed with the use of Agilent Technologies 2100 Bioanalyzer High Sensitivity DNA Chip. Illumina’s NovaSeq 6000 sequencing system (Illumina, San Diego, CA, USA) was used to conduct the 2 × 150 paired-ended sequencing analysis with a minimum sequencing depth of 40 million reads per sample.

### Bioinformatic analysis

To investigate the regulatory mechanisms affected by VIS in the porcine APc, the expression of genes, as well as long noncoding RNAs (lncRNAs) and alternative splicing (AS) events were analysed. The in silico analyses were performed as described in our previous study [40], with modifications. Visualization of the obtained data was conducted using SRplot online tools (http://www.bioinformatics.com.cn/).

### Transcripts assembly and differentially expressed transcripts processing

To remove the reads containing adapters, and low-quality scores (Q_Phred_ score ≤ 20; reads containing more than 5% of unknown nucleotides), Cutadapt (v.1.9) [41] and custom Python scripts were applied. The quality of the obtained raw reads was determined using FastQC (v.0.11.9) [42]. HISAT2 (v.2.0.4) [43] script was applied to map the filtered reads to *Sus scrofa domestica* genome (v.107; [44]) deposited in Ensembl database [45]. The mapped reads were assembled using StringTie (v.1.3.4d) [46]. The transcriptomes from all samples were merged to reconstruct a comprehensive transcriptome using custom Python scripts and gffcompare (v.0.9.8) [47]. Subsequently, StringTie and ballgown [48] were used to estimate the expression levels of all transcripts.

To determine the expression levels for mRNAs and lncRNAs by calculating FPKM (fragments per kilobase of transcript per million fragments mapped), the StringTie script was applied. The differences in expression of mRNAs and lncRNAs between control and VIS-treated groups were determined with DESeq2 [49] software (and edgeR between 2 samples; [50]). Due to the peri-physiological concentration of the used treatment, the expected changes in the genes and lncRNA expression were rather discrete. Therefore, the cutoff level for DEGs or differentially expressed lncRNAs (DELs) was defined at the level of 0.56 (log_2_FC ≥ 0.56). All results with *P* < 0.05 were considered statistically significant.

### Functional annotation of target genes

The gene set enrichment analysis was performed using GSEA software v.4.1.0 [51] and Molecular Signatures Database (MSigDB). Enrichment ontology and pathway analysis were performed based on the Kyoto Encyclopedia of Genes and Genomes (KEGG) [52] and GO [53, 54] databases. The KEGG and GO terms with *P* < 0.05 were defined as significant.

### Interaction network of DEGs

The interaction network between chosen DEGs was generated using the GeneMania Prediction Server [55]. From the list of DEGs with log_2_FC > 0.56, 15 were chosen. The selected gene products were found to be involved in several processes such as the regulation of pituitary functioning, apoptosis or angiogenesis. The interaction networks were generated based on the known interplay between genes: co-expression, genetic interactions, co-localization (*cis*-interactions), physical interactions, shared protein domains and signalling pathways.

### Identification of lncRNAs, target gene prediction and functional analysis

To identify lncRNAs candidates whose expression differed between the porcine APc treated and non-treated with VIS, the transcripts overlapping with known mRNAs, known lncRNAs and transcripts shorter than 200 bp were excluded. The remained sequences were analyzed with the use of a Coding Potential Calculator (CPC) (v.0.9-r2) [56] and a Coding-Non-Coding Index (CNCI) (v.2.0) [57] tools to predict transcripts with coding potential. Transcripts with a CPC score < 0.5 and a CNCI score < 0 were qualified and considered as novel lncRNAs. The remaining transcripts with class codes (i, j, o, u, x) were considered as known lncRNAs. Subsequently, to identify potential *cis* interactions of lncRNAs with the neighbouring genes, a custom Python script was applied to select 100,000 upstream and downstream coding genes. To identify differences in lncRNA sequences’ expression between the probes from control and VIS-treated groups, DESeq2 software was applied. The functional analysis of the target genes for lncRNAs was conducted and the results with a false discovery rate (FDR; *q*) < 0.05 were considered statistically significant. The obtained DELs were descended into one of five categories, according to class code generated by StringTie software [46]: (i) a transfrag falling entirely within a reference intron (intronic); (j) potentially novel isoform or fragment at least one splice junction is shared with a reference transcript; (o) generic exonic overlap with a reference transcript; (u) unknown, intergenic transcript (intergenic); and (x) exonic overlap with reference on the opposite strand (antisense). The relatively close localization of DELs and DEGs on the same chromosome (up to 100,000 bp) were qualified into the category *cis*-interactions.

### Alternative splicing analysis

To identify AS events, as well as to analyze differential alternative splicing events (DASs) between control and VIS-treated groups, a replicate multivariate analysis of transcript splicing (rMATS) (v.4.1.1) script was applied [51]. Trimmed reads with 120 bp length were applied to estimate the percent of splicing inclusion (PSI) in a range of splicing sites at intron/exon junctions. AS events which passed the criteria of *q* < 0.05 and the absolute value of inclusion level difference, |ΔPSI| > 0.1 were considered statistically significant. The revealed DASs were classified into one of five alternative splicing types, as follows: alternative 3′ splice site (A3), 5′ splice site (A5SS), mutually exclusive exons (MXE), retention intron (RI), and skipping exon (SE).

### Validation of DEGs and DELs by quantitative real-time PCR (qPCR) method

To validate the results obtained from NGS, qPCR analysis was conducted. The analysis was conducted using Aria Mx Real-time PCR System (Agilent Technology, USA) as described by Smolinska et al. [58], with the use of the same RNA as in the RNAseq method (*n* = 5/group). Specific primer pairs used to amplify parts of 5 chosen DEGs: CD180 molecule (*CD180*), inhibin subunit beta E (*INHBE*), regucalcin (*RGN*), midkine (*MDK*) and angiogenin (*ANG*), as well as for 2 chosen DELs: *ENSSSCT00000101418* and *ENSSSCT00000069084.* The following reference genes were used: ubiquitin C (*UBC*), and 18S ribosomal RNA (*18sRNA*). Primer sequences, as well as qPCR reaction conditions, were detailed in Table 1. The constitutively expressed genes *UBC* and *18sRNA* were used as the internal controls to verify the method. The reaction mixture contained the following components: cDNA, primers, Sensitive RT HS-PCR Mix SYBR (12.5 µL), and ROX (0.24 µL; reference dye), along with RNase-free water to a final volume of 20 µL. The negative non-template controls (NTCs) contained water instead of cDNA, or the reverse transcription step was omitted. All reactions were run in duplicates. To confirm the specificity of qPCR reactions, at the end of the experiment, the analysis of the melting curve was carried out. The purity of the products was confirmed with the use of agarose gel electrophoresis. The calculation of validated DEGs/DELs relative expression was conducted with the use of the comparative cycle threshold method (ΔΔCT) and normalized using the geometrical means of the reference genes’ Ct values [59]. The normality of qPCR data distributions was confirmed by the Shapiro–Wilk test (*P* > 0.05). The results of the experiments were analyzed with the use of a Student *t*-test using Statistica software (Statsoft Inc., USA). Values for *P* < 0.05 were considered statistically significant.

**Table 1:** Primers, qPCR and RT-PCR reaction conditions used for the validation of the obtained results

Primer target	Primer sequence (5′→3′)	Accession number		Amplicon size, nt	Primer, nmol/L	Conditions	Reference
Differentially expressed genes (DEGs)							
* CD180*	F: CCTAAGCCACAACAGCCTGA R: ATTCGAGCAAGTGCAGTCCA	NM_214357.1	178		400	Activation: 95 ºC – 10 min; 40 cycles of: Denaturation: 95 ºC – 15 s Annealing: 60 ºC – 1 min Elongation: 72 ºC – 1 min	This study
* INHBE*	F: CCTTGTGCACCGTAGGCGGG R: GGCTTGCCAGGCCCCAAGAG	XM_003126320.4	191		400	Activation: 95 ºC – 10 min; 40 cycles of: Denaturation: 95 ºC – 15 s Annealing: 60 ºC – 1 min Elongation: 72 ºC – 1 min	This study
* RGN*	F: CCAAACCGTGAAGTTGCCTG R: 5’GGTTGCTGCAAAAGACCCTG	NM_001077220.1	123		400	Activation: 95 ºC – 10 min; 40 cycles of: Denaturation: 95 ºC – 15 s Annealing: 60 ºC – 1 min Elongation: 72 ºC – 1 min	This study
* MDK*	F: CCGGCAGAGAGCGAGATG R: GCAGTCAGCTCCAAACTCCT	NM_001195352.1	267		400	Activation: 95 ºC – 10 min; 40 cycles of: Denaturation: 95 ºC – 15 s Annealing: 60 ºC – 1 min Elongation: 72 ºC – 1 min	This study
* ANG*	F: CCCCTGCTGTTGGTCTTCAT R: TGGTTTGGCATCGTAGTGCT T3	NM_001044573.2	108		400	Activation: 95 ºC – 10 min; 40 cycles of: Denaturation: 95 ºC – 15 s Annealing: 57 ºC – 1 min Elongation: 72 ºC – 1 min	This study
* UBC*	F: GGAGGAATCTACTGGGGCGG R: CAGAAGAAACGCAGGCAAACT	XM_003483411.3	103		400	Activation: 95 ºC – 10 min; 40 cycles of: Denaturation: 95 ºC – 15 s Annealing: 60 ºC – 1 min Elongation: 72 ºC – 1 min	[138]
* 18sRNA*	F: TCCAATGGATCCTCGCGGAA R: GGCTACCACATCCAAGGAAG	AY265350.1	149		400	Activation: 95 ºC – 10 min; 40 cycles of: Denaturation: 95 ºC – 15 s Annealing: 60 ºC – 1 min Elongation: 72 ºC – 1 min	[139]
Differentially expressed long noncoding RNA (DELs)							
* ENSSSCT00000101418*	F: 5CAGTGCTGCATGGGAACCTA R: 5AAGGGGCAAAACCACATTGC	ENSSSCT00000101418.1	144		400	Activation: 95 ºC – 10 min; 40 cycles of: Denaturation: 95 ºC – 15 s Annealing: 65 ºC – 1 min Elongation: 72 ºC – 1 min	This study
* ENSSSCT00000069084*	F: CTGTGGAGCCTGGAAGAACC R: TCTGAGTCCCCTTTTGCACC	ENSSSCT00000069084.2	198		400	Activation: 95 ºC – 10 min; 40 cycles of: Denaturation: 95 ºC – 15 s Annealing: 63 ºC – 1 min Elongation: 72 ºC – 1 min	This study
Differentially expressed alternative splicing events (DASs)							
* GPR173*	F: TCCTAGGTGAGACAGCGTGA R: CCGTGCTCTTTTGCTTCCAC	ENSSSCT00000099213.1	Skipping: 585 Inclusion: 672		600	Activation: 95 ºC – 10 min; 40 cycles of: Denaturation: 95 ºC – 15 s Annealing: 65 ºC – 1 min Elongation: 72 ºC – 1 min	This study
* MSL1*	F: GCACCCATCCCAAGGAGAAA R: GTGTGTTTTCGAAGCGGCAT	ENSSSCT00000069296.1	Skipping: 252 Inclusion: 299		600	Activation: 95 ºC – 10 min; 40 cycles of: Denaturation: 95 ºC – 15 s Annealing: 67 ºC – 1 min Elongation: 72 ºC – 1 min	This study
* CFLAR*	F: CCACGGAGTGTTTCACCTCA R: TCCAGTCAACAGAAGCCCAC	ENSSSCT00000036156.4	Skipping: 115 Inclusion: 286		600	Activation: 95 ºC – 10 min; 40 cycles of: Denaturation: 95 ºC – 15 s Annealing: 62 ºC – 1 min Elongation: 72 ºC – 1 min	This study
* MAPK6*	F: TGCCCAAGTTGAGTCTCGC R: GCTCCAGCTCACCACAATCA	ENSSSCT00000050785.2	Skipping: 180 Inclusion: 224		600	Activation: 95 ºC – 10 min; 40 cycles of: Denaturation: 95 ºC – 15 s Annealing: 69 ºC – 1 min Elongation: 72 ºC – 1 min	This study
* PI4KA*	F: CATGTACCCACCACACCCTC R: GGAGATCTTCTGGCCATCGG	ENSSSCT00000011053.5	Skipping: 598 Inclusion: 652		600	Activation: 95 ºC – 10 min; 40 cycles of: Denaturation: 95 ºC – 15 s Annealing: 65 ºC – 1 min Elongation: 72 ºC – 1 min	This study

*CD180* CD180 molecule, *INHBE* Inhibin subunit beta E, *RGN* Regucalcin, *MDK* Midkine, *ANG* Angiogenin, *UBC *Ubiquitin C, 1*8sRNA* 18S ribosomal RNA, *GPR173 *G protein-coupled receptor 173, *MSL1 *MSL complex subunit 1, *CFLAR *CASP8 and FADD like apoptosis regulator, *MAPK6 *Mitogen-activated protein kinase 6, *PI4KA *Phosphatidylinositol 4-kinase alpha, *F* Forward, *R* Reverse

### Polymerase chain reaction (RT-PCR; DASs validation)

The characteristics of SE events in 5 chosen genes: G protein-coupled receptor 173 (*GPR173*), MSL complex subunit 1 (*MSL1*), CASP8 and FADD-like apoptosis regulator (*CFLAR*), mitogen-activated protein kinase 6 (*MAPK6*), and phosphatidylinositol 4-kinase alpha (*PI4KA*), were conducted with the use of Labcycler 48s (Syngen Biotech, Poland) and StartWarm HS-PCR Mix (A&A Biotechnology, Poland). The reaction mixture (in a final volume of 25 µL) contained 12.5 µL of Hot Start PCR Mix, forward and reverse primers, nuclease-free deionized water, and cDNA. NTCs were prepared as described in the previous section. PCR conditions and primers’ concentrations were determined experimentally in a temperature gradient and, together with the primer sequences, are detailed in Table 1. The obtained PCR products were analysed on 1.5% agarose gels containing Midori Green Advance dye (Nippon Genetics Europe, Germany).

## Results

### Quality control and statistics of reads

A total of 479,237,334 raw paired-end reads contained an average of 47,923,733 reads per sample revealed. The total alignment to the *Sus scrofa* gave 48%–77% of coverage. The average number of 47.92 million valid reads, and 33.24 million mapped reads passed the Illumina producers’ criteria allowing for enriching the present study with DASs analysis. On average, 68.68% of reads were mapped uniquely, whereas 1.09% were found to be mapped in multiple loci. About 61.54% of the obtained reads were mapped to coding DNA sequence regions (CDS), whereas the rest belonged to introns (34.8%) or intergenic regions (3.66%). The percentage of uniquely mapped reads results from the adopted method of constructing sequencing libraries (ribo-zero), and the high percentage of nucleotides mapped to CDS regions confirm a high proportion of coding transcripts in the obtained reads. The relatively high percentage of nucleotides mapped to introns indicated the chance of detecting large changes in intron retention type of alternative splicing. Taking into consideration the applied library constructing method, a large part of lncRNA coding sequences was automatically assigned to the intron group at the first step of the analysis. The lncRNA sequences were separated at the further steps of analyses. The correctness of the results for the obtained readings was confirmed in the validation process described below. All the statistics for the individual samples have been detailed in Supplementary File 1. Raw reads were deposited in the Functional Genomics Data Collection (ArrayExpress) database under the common project accession number E-MTAB-13,922.

### DEGs and functional annotations (GO; KEGG)

**Fig. 1:**
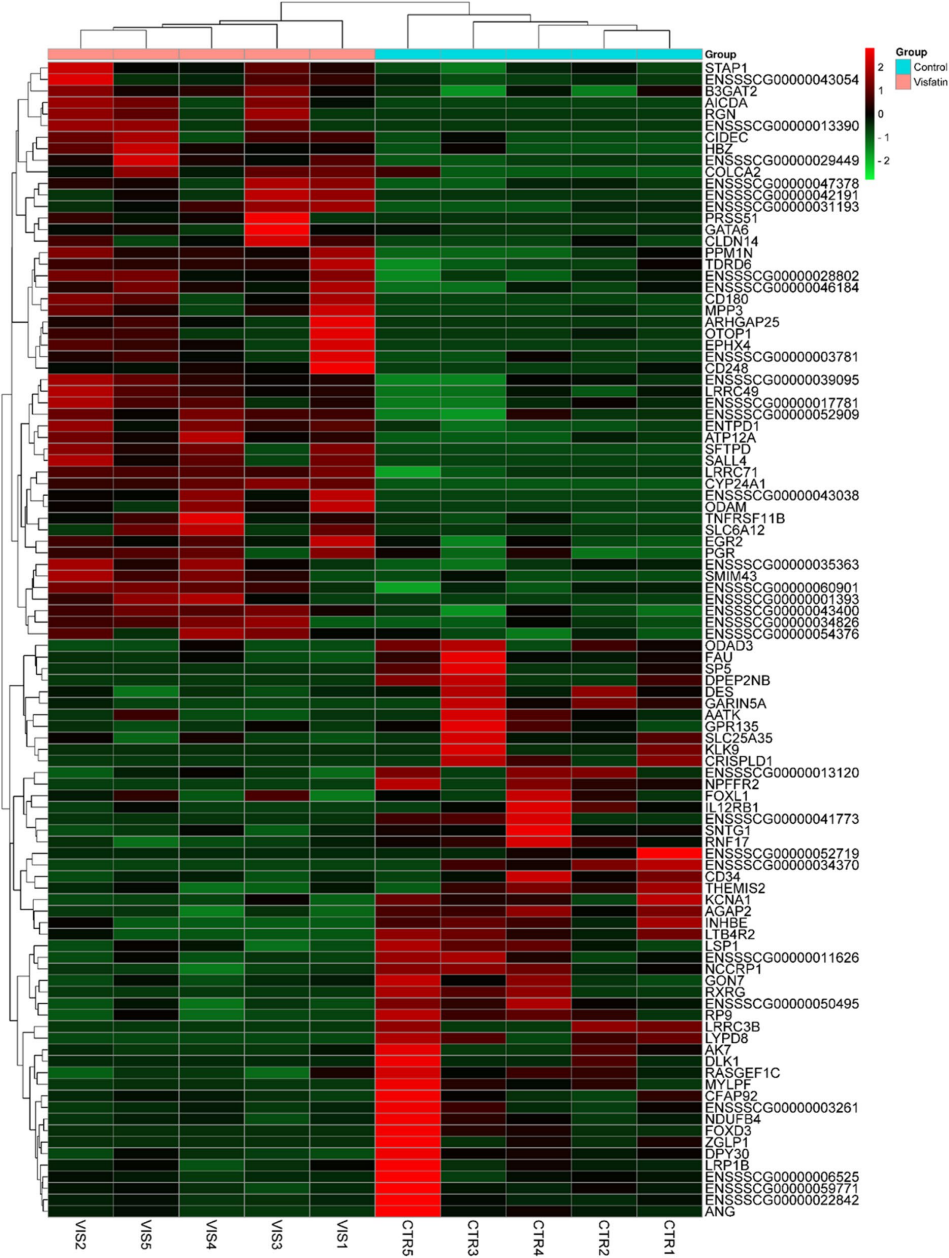
The hierarchical clustering heatmap of chosen genes whose expression was altered under the influence of visfatin. Each column represents biological replicates of control (CTR1-5) or visfatin (VIS1-5). Different colours of brackets represent the normalized (Z-score; red-green scale) expression values for DEGs in each biological replicate

**Fig. 2:**
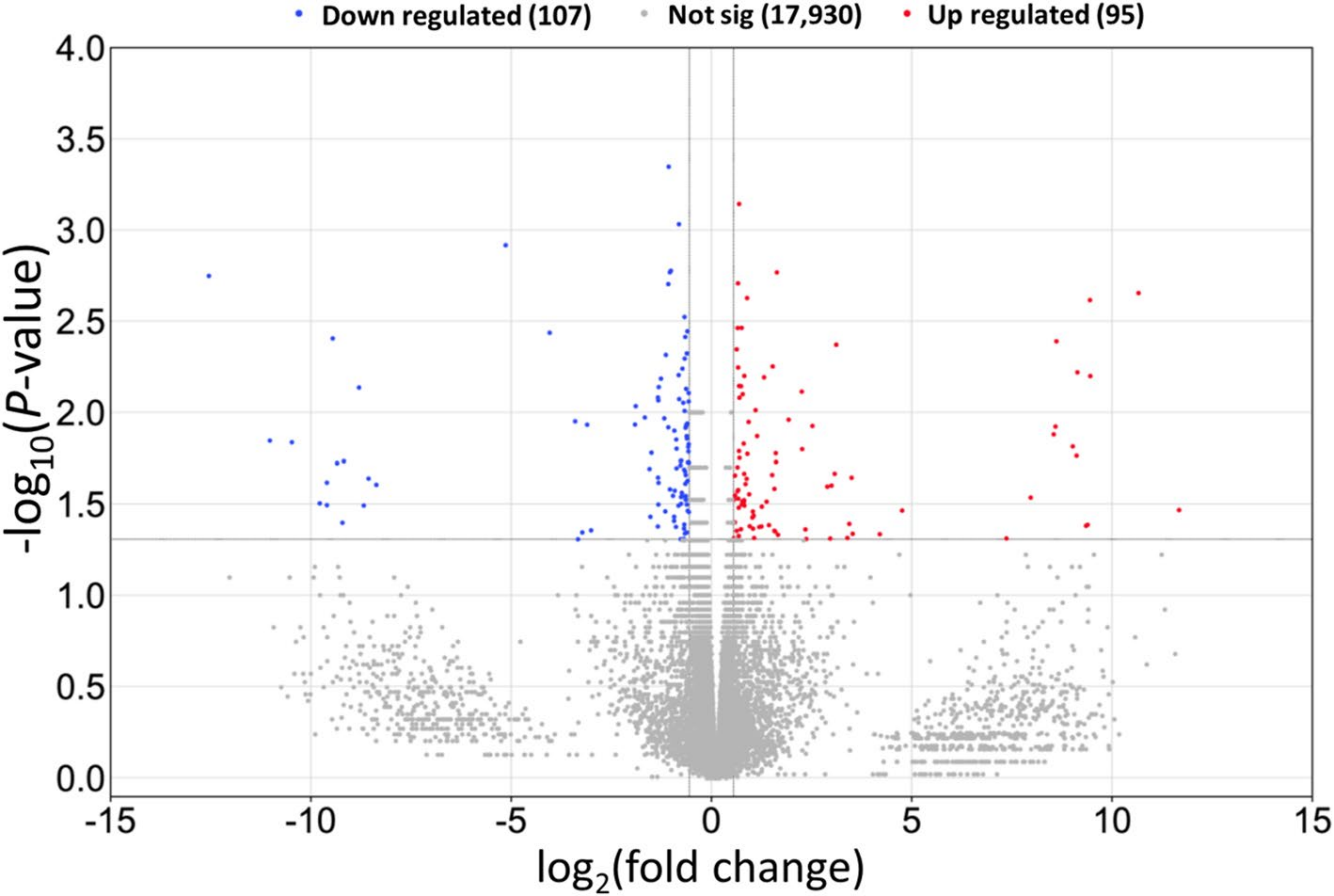
Visualization of the number of genes whose expression changed in the presence of visfatin. The *X*-axis represents logarithmic fold changes in expression (log_2_FC), whereas the *Y*-axis shows the negative decimal logarithm of the *P*-values. The horizontal line refers to the negative logarithmic *P*-value cut-off ( *P*  = 1.3). The vertical lines mark the fold change cut-offs (log_2_FC > |0.56|)

**Fig. 3:**
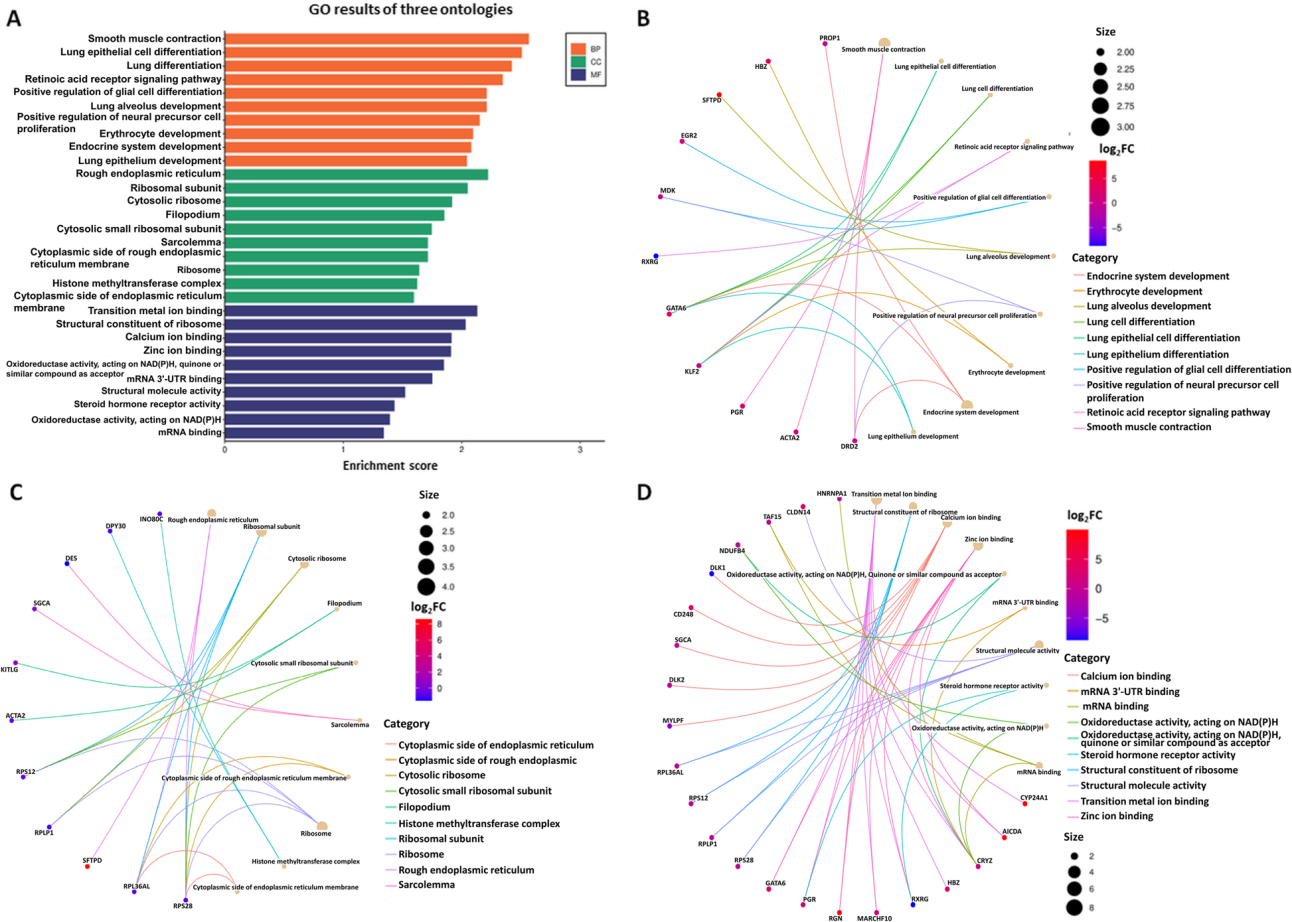
Results of Gene Ontology (GO) analysis of genes whose expression differed under the influence of visfatin (*P* < 0.05, log_2_FC > |0.56|). **A** Bar plot of GO components enriched by DEGs evaluated in the anterior pituitary cells treated with visfatin. Orange bars represent GO terms connected with biological processes (BP). Green bars represent GO terms related to the cellular component (CC) category. Dark blue bars represent GO terms connected with the molecular function (MF) category. The vertical axis contains the names of GO terms. The horizontal axis describes the enrichment score based on the number of significant DEGs. Net plots present the GO enrichment of processes connected with BP (**B**), CC (**C**) and MF (**D**)categories. Different colour lines mean different GO terms. Different size of the dots reflects the number of significant DEGs assigned to GO terms. The colour of the dots reflects changes in the expression of the involved DEGs (log_2_FC)

**Fig. 4:**
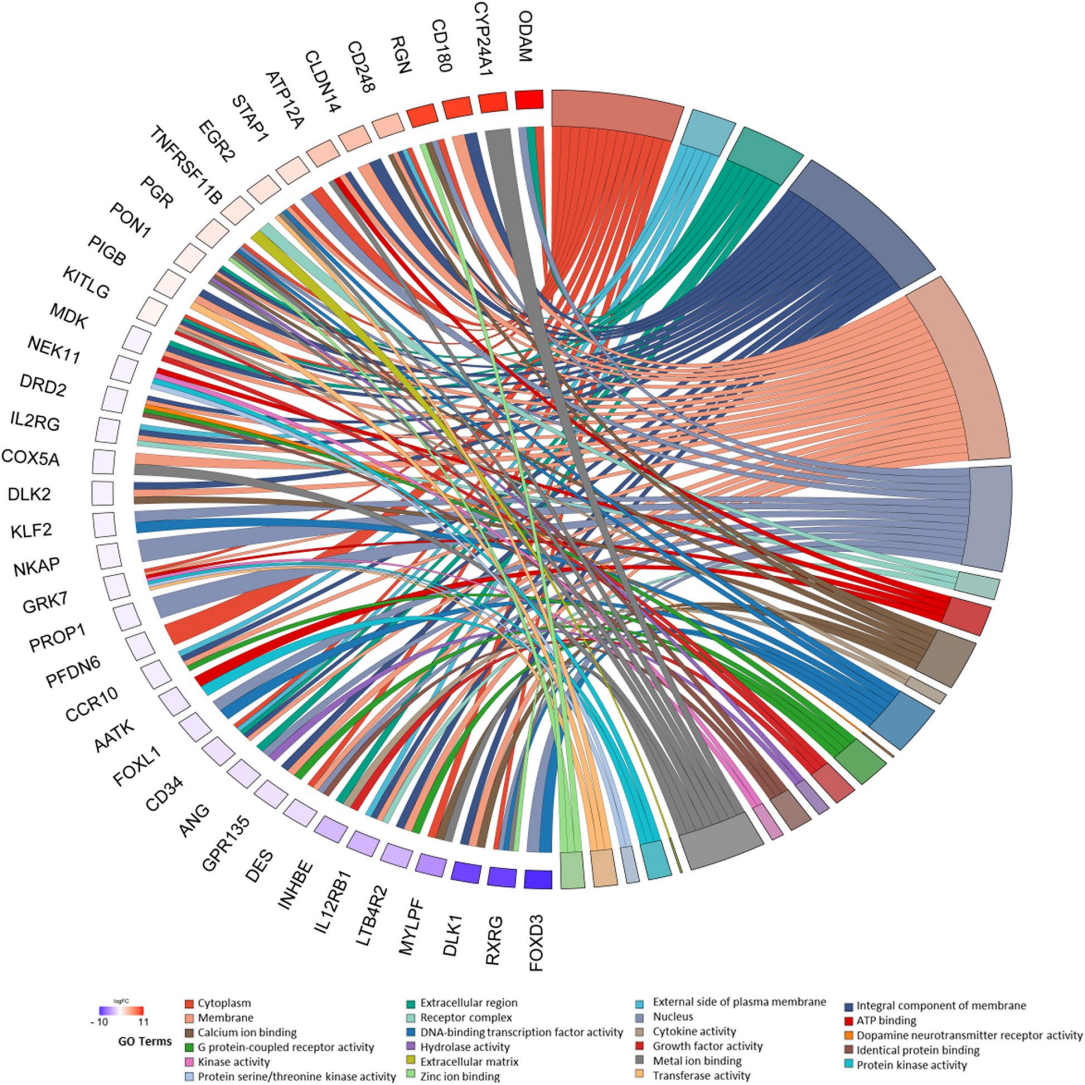
The visualization of the statistically significant, differentially expressed genes (DEGs) and their enrichment in ontology terms. Circos plot presents selected Gene Ontology terms associated with porcine anterior pituitary cells’ DEGs with expression altered under the influence of visfatin ( *P*  < 0.05, log_2_FC>|0.56|). Different colours of lines mean different GO terms, whereas the colour of bars assigned to the genes’ symbols represents the ratio between up- and downregulated genes. Red bars represent GO terms with the predominance of upregulated DEGs, and blue bars show terms with a predominance of downregulated DEGs

Out of a total of 18,132 identified genes, 202 met the criteria of log_2_FC >|0.56| and *P* < 0.05, and have been presented in Fig. 1 and 2. Among all 202 obtained DEGs, 95 were upregulated and 107 were downregulated under the influence of VIS (Supplementary File 2). To discover possible functions of DEGs identified in the APc treated with VIS, the genes were classified into one of the following GO categories: ‘biological processes’ (BP), ‘cellular components’ (CC) and ‘molecular function’ (MF). DEGs were assigned to 318 GO terms (*P* < 0.05). Among them, 208 terms were assigned to BP, 44 to CC and 66 to MF category (Supplementary File 3). In the BP category, the most of DEGs were connected with *positive regulation of transcription by RNA polymerase II* (GO:0045944; 29 DEGs), *negative regulation of transcription by RNA polymerase II* (GO:0000122; 26 DEGs) and *translation* (GO:0006412; 22DEGs). In the CC category, the most enriched terms were *nucleus* (GO:0005634; 116 DEGs), *ribosome* (GO:0005840; 23 DEGs) and *chromatin* (GO:0000785; 15 DEGS). In the MF category, the biggest number of DEGs was related to *DNA binding* (GO:0003677; 38 DEGs), *RNA polymerase II cis-regulatory region sequence-specific DNA binding* (GO:0000978; 26 DEGs), *structural constituent of ribosome* (GO:0003735; 21 DEGs) and *sequence-specific DNA binding* (GO:0043565; 20 DEGs). The results of GO analysis have been presented in Fig. 3. Figure 4 presents the enrichment of GO terms with chosen, important DEGs.

**Fig. 5:**
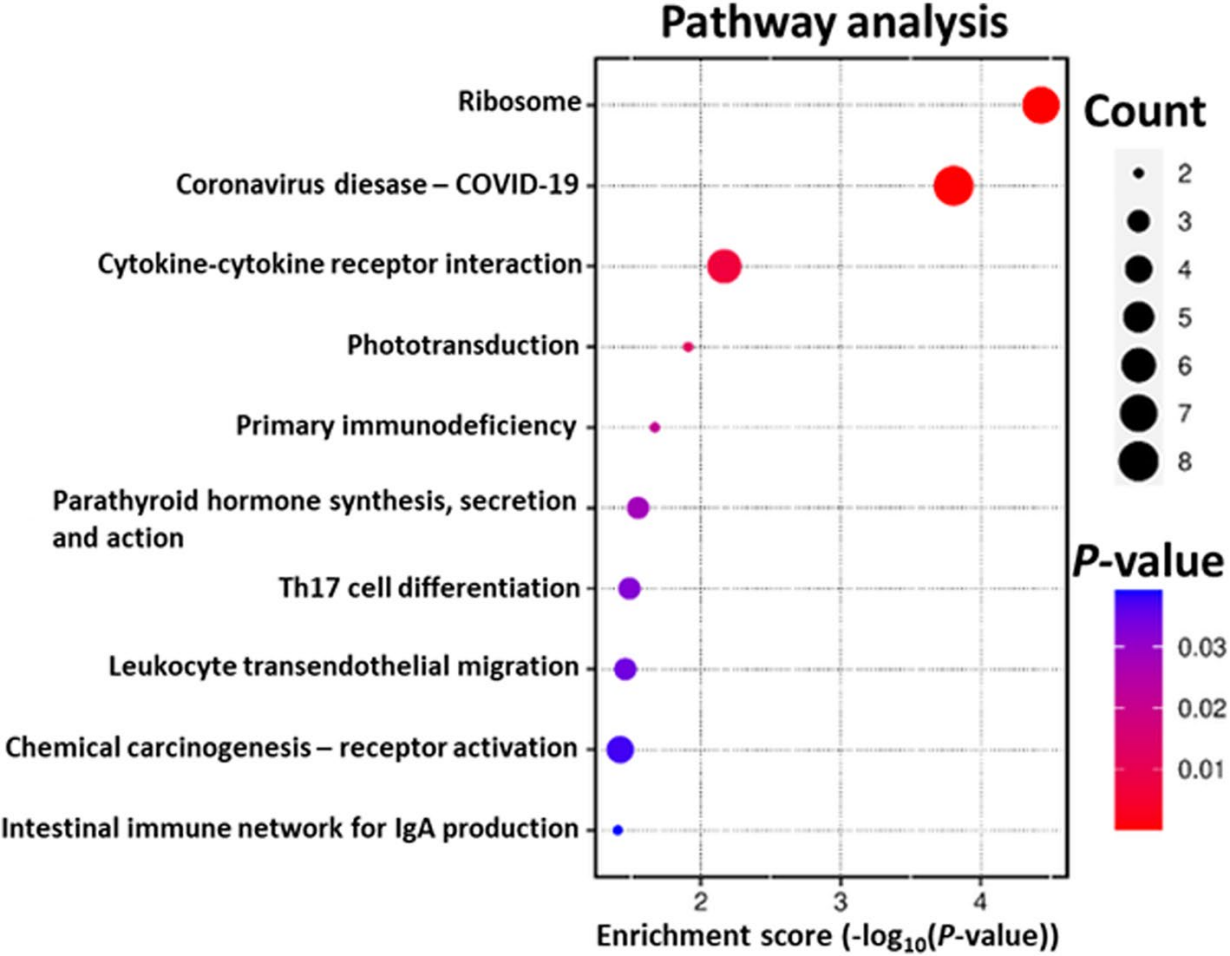
Scatterplot diagram presenting the enrichment of KEGG pathways with DEGs under the influence of visfatin (*P* < 0.05, log_2_FC > |0.56|). The vertical axis describes the revealed pathways. The horizontal axis describes the enrichment score calculated based on the negative decimal logarithm of the pathway’s *P*-value. The size of the dots reflects the number of DEGs involved in the pathway

The analysis of KEGG database revealed 16 signalling pathways (*P* < 0.05), from which the most enriched were those connected with *Coronavirus disease-COVID-19* (ko05171;19 DEGs), *Ribosome* (ko03010; 18 DEGs), *Amyotrophic lateral sclerosis* (ko05014; 15 DEGs) and *Pathways of neurodegeneration - multiple diseases* (ko05022; 15 DEGs). The results of the KEGG databases analysis have been presented in Fig. 5. The detailed results of the analysis have been attached in Supplementary File 3.

### Interaction networks between DEGs

**Fig. 6:**
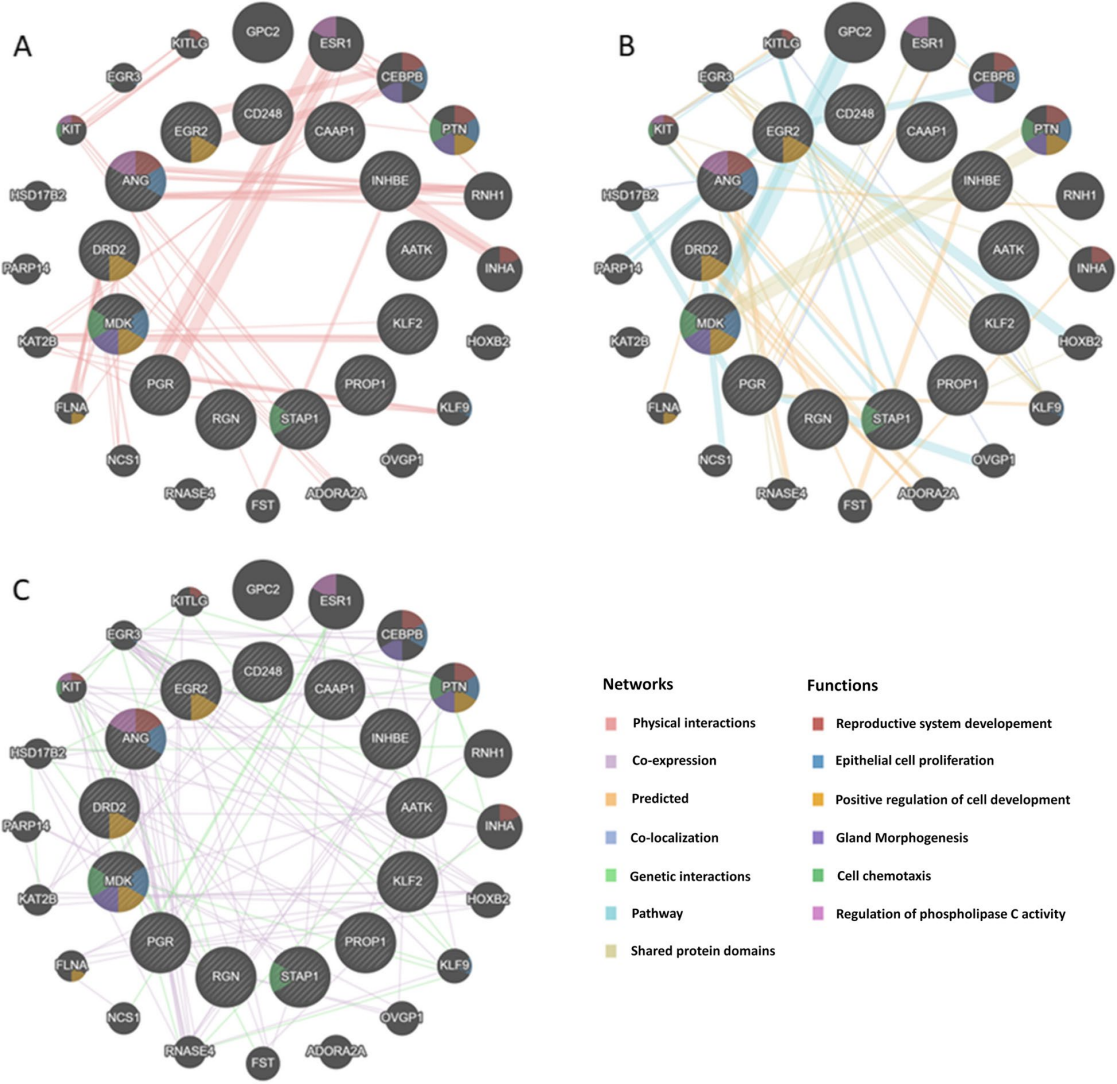
Gene interaction network developed in GeneMania for selected genes; the colour of a line connecting genes denotes the type of interaction, and the colour of the circle denotes the gene’s product function (refer to the key)

The interaction network was created between 34 chosen DEGs, involved in, i.a., positive regulation of cell-cell adhesion, tissue remodelling, cytokine activity, interleukin-6 production, leukocyte migration, angiogenesis and embryonic hemopoiesis. GeneMania was used to predict the relations between the chosen genes (query genes) in seven different types of interaction as follows: physical interactions (Fig. 6A), co-expression and genetic interactions (Fig. 6B), as well as co-localization, pathways, predicted and shared protein domains (Fig. 6C). The analysis considered an additional 20 automatically generated genes, which were necessary to indicate the observed networks (interacting genes). Moreover, using different colours, we indicated the contribution of genes in specific biological functions: reproductive system development (red), epithelial cell proliferation (blue), positive regulation of cell development (orange), gland morphogenesis (purple), cell chemotaxis (green) and regulation of phospholipase C activity (light purple; refer to the key in Fig. 6). Co-expression of the genes was found in 99 interactions and co-localization was observed in 5 interactions. In 15 cases, the genes participated in common pathways. Genetic interaction was revealed in 28 cases. Physical interactions were present in 59 cases, and in 23 cases interactions were based on the shared protein domains. In 18 cases, the interaction between genes was assigned to the ‘predicted’ category. The complete list of gene interactions is presented in Supplementary File 6.

### Identification of lncRNAs, target gene prediction and functional analysis

Out of the total number of 11,204 identified lncRNA candidates, 79 passed the criteria of FDR < 0.05 and log_2_FC > 0.56 and were qualified as DELs. Out of them, 44 were annotated with ENSEMBL gene IDs, and the other 34 were predicted during the procedure for identifying novel lncRNAs. In the obtained group of DELs, 47 were upregulated, whereas 32 were downregulated, in the VIS-treated group (Table 2). The analysis revealed 83 interactions. Among them, 63 results indicated the colocalization in a range of 100,000 bp, 15 were in close proximity (10,000 bp) and 3 were in very close proximity (1,000 bp). Only four of the revealed lncRNAs showed a high correlation with the target gene (Pearson Correlation Coefficient > 0.8), however, their target genes did not pass the criteria for DEGs. Because we did not identify any significant DEL–DEG pairs, data on the protein-coding genes and DELs co-localization were not included in further functional analyses and the following GO analysis was conducted for genes connected with DELs, whose did not fulfil the DEGs definition.

**Table 2:** List of differentially expressed lncRNAs revealed in the porcine anterior pituitary cells subjected to the influence of visfatin during the mid-luteal phase of the oestrous cycle

Transcript ID	Reference gene ID	log_2_(FC)	adj. *P*	Reg	Chr	Str	Start—End
MSTRG.7664.3	E*G00000048856	16.58	1.65E–19	Up	chr2	–	6,750,269–6,757,189
MSTRG.3089.2	E*G00000052388	16.44	2.82E–15	Up	chr12	–	34,885,637–34,899,596
MSTRG.13770.7	E*G00000036924	15.65	1.03E–03	Up	chr7	–	47,442,821–47,448,891
MSTRG.13080.4	E*G00000036505	15.01	7.90E–17	Up	chr6	+	144,782,510–144,918,863
MSTRG.9740.5	E*G00000008440	14.89	2.65E–16	Up	chr3	+	93,951,147–93,973,622
MSTRG.2806.3	E*G00000032176	14.14	7.23E–18	Up	chr12	–	14,361,127–14,430,774
MSTRG.14086.4	E*G00000037267	14.14	1.07E–13	Up	chr7	–	89,143,309–89,144,497
MSTRG.2806.8	E*G00000032176	13.59	1.25E–16	Up	chr12	–	14,387,858–14,398,586
E*T00000078307	E*G00000050540	12.71	2.29E–13	Up	chr3	+	23,119,351–23,122,822
E*T00000084922	E*G00000048633	11.88	2.43E–13	Up	chr2	+	6,214,619–6,219,110
MSTRG.3584.2	E*G00000038362	11.74	1.62E–12	Up	chr13	–	18,461,958–18,485,526
E*T00000069582	E*G00000050595	10.27	1.49E–11	Up	chr2	+	116,257,556–116,277,804
MSTRG.13886.2	E*G00000029776	10.23	1.85E–11	Up	chr7	+	58,654,894–58,658,906
E*T00000078089	E*G00000042082	10.15	8.75E–07	Up	chr1	+	17,142,455–17,145,258
MSTRG.15282.3	E*G00000055798	9.83	1.45E–11	Up	chr9	+	60,467,366–60,472,719
E*T00000074057	E*G00000048558	8.94	4.51E–05	Up	chr13	–	21,667,242–21,670,382
E*G00000059923	E*T00000099576	8.68	2.67E–04	Up	chr8	–	18,102,710–18,105,972
E*T00000098638	E*G00000061319	8.18	8.99E–05	Up	chr17	–	2,006,944–2,011,058
MSTRG.126.3	E*G00000061323	7.20	1.36E–04	Up	chrX	–	40,571,101–40,596,610
E*T00000105718	E*G00000059333	4.47	4.49E–04	Up	chr11	+	9,792,503–9,795,311
E*T00000100497	E*G00000060452	4.36	6.07E–05	Up	chr13	–	119,692,922–119,698,876
E*T00000101371	E*G00000061444	4.05	3.92E–04	Up	chr11	+	7,474,385–7,476,826
E*T00000093905	E*G00000060577	3.44	8.07E–04	Up	chr13	–	82,442,292–82,548,411
MSTRG.126.8	E*G00000061762	3.44	2.58E–04	Up	chrX	–	40,582,533–40,598,330
E*T00000098236	E*G00000054408	3.34	1.22E–03	Up	chr2	–	28,880,473–28,973,419
E*T00000101696	E*G00000053327	2.94	1.89E–04	Up	chr15	–	2,326,404–2,330,887
E*T00000098343	E*G00000052782	2.79	2.15E–04	Up	chr11	–	47,513,188–47,519,300
E*T00000104568	E*G00000062124	2.46	2.12E–04	Up	chr2	+	118,185,204–118,189,197
E*T00000092251	E*G00000058643	1.99	5.41E–04	Up	chr7	–	43,734,725–43,737,757
E*T00000095926	E*G00000058334	1.34	6.31E–05	Up	chr7	–	12,657,382–12,665,985
E*T00000104974	E*G00000061904	1.23	4.14E–04	Up	chr3	+	97,129,724–97,134,442
E*T00000096838	E*G00000058457	1.08	7.17E–04	Up	chr13	+	66,179,114–66,186,129
E*T00000085618	E*G00000043222	1.06	1.15E–03	Up	chr14	–	28,607,291–28,609,501
E*T00000094869	E*G00000061905	0.94	1.60E–04	Up	chr16	+	6,128,683–6,133,531
E*T00000069191	E*G00000016026	0.89	2.13E–04	Up	chr15	+	91,526,109–91,529,667
E*T00000101889	E*G00000059224	0.84	3.65E–05	Up	chr3	+	59,493,139–59,497,205
E*T00000093850	E*G00000053608	0.83	1.63E–04	Up	chr8	+	55,508,455–55,512,425
E*T00000094652	E*G00000061365	0.81	7.66E–09	Up	chr1	+	25,765,864–25,771,305
MSTRG.9517.1	E*G00000008136	0.79	1.26E–04	Up	chr3	.	47,598,992–47,599,312
E*T00000085660	E*G00000046432	0.71	4.16E–04	Up	chr16	+	34,141,063–34,145,966
E*T00000098645	E*G00000058146	0.64	4.78E–04	Up	chr6	–	15,867,883–15,872,637
E*T00000094789	E*G00000056401	0.63	2.26E–04	Up	chr3	+	59,551,195–59,554,319
MSTRG.5145.1	E*G00000010107	0.60	7.18E–05	Up	chr14	.	50,739,497–50,740,520
E*T00000093477	E*G00000057944	0.59	2.33E–04	Up	chr11	–	52,147,543–52,152,640
MSTRG.3250.1	E*G00000028720	0.59	2.36E–07	Up	chr12	–	46,915,213–46,954,164
E*T00000069084	E*G00000048792	0.59	1.17E–03	Up	chr16	–	50,421,817–50,426,340
MSTRG.3252.1	E*G00000028720	0.57	1.26E–04	Up	chr12	–	46,984,331–47,019,831
E*T00000101418	E*G00000054768	–1.39	1.15E–03	Down	chr2	+	15,673,197–15,676,332
MSTRG.13156.1	N/A	–2.25	1.17E–03	Down	chr6	.	157,890,455–157,890,709
E*T00000072255	E*G00000048152	–2.4	3.36E–05	Down	chr7	–	53,563,422–53,571,908
E*T00000099215	E*G00000059569	–2.69	1.53E–05	Down	chr1	–	108,983,443–108,986,978
E*T00000092543	E*G00000060701	–4.59	8.42E–04	Down	chr13	+	125,743,165–125,747,322
E*T00000093959	E*G00000055538	–7.3	1.25E–03	Down	chrX	–	75,804,372–75,810,968
MSTRG.8629.1	E*G00000041901	–8.33	3.27E–09	Down	chr2	.	119,570,655–119,570,955
MSTRG.481.1	N/A	–8.63	2.95E–09	Down	chrY	.	25,272,845–25,273,118
MSTRG.480.1	N/A	–8.76	1.30E–09	Down	chrY	.	25,261,737–25,261,979
MSTRG.13391.1	N/A	–9.04	1.23E–10	Down	chr7	.	10,867,702–10,868,344
MSTRG.3977.1	E*G00000011580	–9.38	1.57E–11	Down	chr13	.	68,477,907–68,478,124
MSTRG.2806.9	E*G00000032176	–9.75	6.05E–13	Down	chr12	–	14,401,555–14,411,927
MSTRG.4884.1	E*G00000009796	–9.78	3.52E–11	Down	chr14	.	30,530,530–30,530,914
MSTRG.7799.5	E*G00000059500	–10.21	2.18E–13	Down	chr2	+	13,149,141–13,191,534
E*T00000095905	E*G00000053497	–10.29	3.03E–13	Down	chr6	–	61,574,889–61,579,423
MSTRG.2523.1	N/A	–10.52	4.77E–12	Down	chr11	.	48,317,432–48,317,639
E*T00000102280	E*G00000056542	–10.98	2.15E–12	Down	chr6	–	82,858,134–82,861,523
MSTRG.4908.4	E*G00000009815	–11.31	4.11E–13	Down	chr14	+	31,363,931–31,366,499
E*T00000085095	E*G00000050540	–11.43	3.45E–14	Down	chr8	+	73,970,667–74,007,774
E*T00000097463	E*G00000051823	–11.79	2.31E–15	Down	chr6	+	6,921,453–6,927,951
E*T00000104080	E*G00000053011	–12.83	2.37E–15	Down	chr10	+	54,260,300–54,263,660
MSTRG.3089.4	E*G00000052388	–13.56	5.45E–14	Down	chr12	–	34,885,709–34,903,165
MSTRG.5383.2	E*G00000010400	–13.61	3.89E–14	Down	chr14	–	90,533,125–90,582,765
E*T00000091734	E*G00000054873	–13.78	1.03E–07	Down	chr6	–	92,220,679–92,225,794
E*T00000051884	E*G00000027988	–13.8	3.86E–06	Down	chr14	+	140,335,875–140,357,762
MSTRG.2507.4	E*G00000040073	–13.83	3.14E–15	Down	chr11	–	45,001,815–45,018,241
E*T00000093481	E*G00000042281	–13.88	3.03E–04	Down	chr18	+	18,005,836–18,011,203
E*T00000094901	E*G00000059465	–14.52	1.20E–20	Down	chr3	+	44,827,873–45,007,886
MSTRG.7311.3	E*G00000016583	–14.85	1.32E–15	Down	chr18	–	19,929,048–19,947,080
MSTRG.13505.3	E*G00000024259	–15.18	3.90E–04	Down	chr7	+	22,633,880–22,637,444
MSTRG.3089.7	E*G00000061739	–15.37	1.49E–15	Down	chr12	–	34,888,165–34,903,165
MSTRG.2900.1	E*G00000063179	–15.67	1.01E–14	Down	chr12	–	19,714,679–19,725,870

*Chr* Chromosome number, ‘E*’ ‘ENSSSC’, *MSTRG *String tie’s gene identifier, *log*_*2*_*(FC)* Binary logarithm of fold change, *N/A *Not available, *adj. P* Adjusted *P*-value, *Str *Strand, ‘+’ Sense strand, ‘−’ Antisense strand, ‘.’  Annotations that are not stranded, *‘down’ *Down-regulation of the expression, *‘up’* Upregulation of the expression

The GO analysis of the obtained results revealed 64 processes in which genes connected with the identified lncRNAs were involved. Among them, 45 were related to the BP cluster, 7 to CC, and 11 to MF. One of the revealed processes, *transport across blood-brain barrier* (GO:0150104; 2 DELs), has not been categorized. In the BP category the most enriched processes were the *defense response to virus* (GO:0051607; 13 DELs), *defense response* (GO:0006952; 11 DELs), *B cell differentiation* (GO:0030183; 10 DELs) and *adaptive immune response* (GO:0002250; 10 DELs). The most enriched processes in the CC category were *cell cortex* (GO:0005938; 10 DELs), *stress fiber* (GO:0001725; 6 DELs) and *endocytic vesicle* (GO:0030139; 6 DELs). The most enriched positions in the MF function were *RNA polymerase II transcription regulatory region sequence-specific DNA binding* (GO:0000977; 20 DELs), *cytokine receptor binding* (GO:0005126; 9 DELs) and *type I interferon receptor binding* (GO:0005132; 8 DELs). The GO analysis results of DELs were detailed in Supplementary File 4.

### Alternative splicing analysis results

**Fig. 7:**
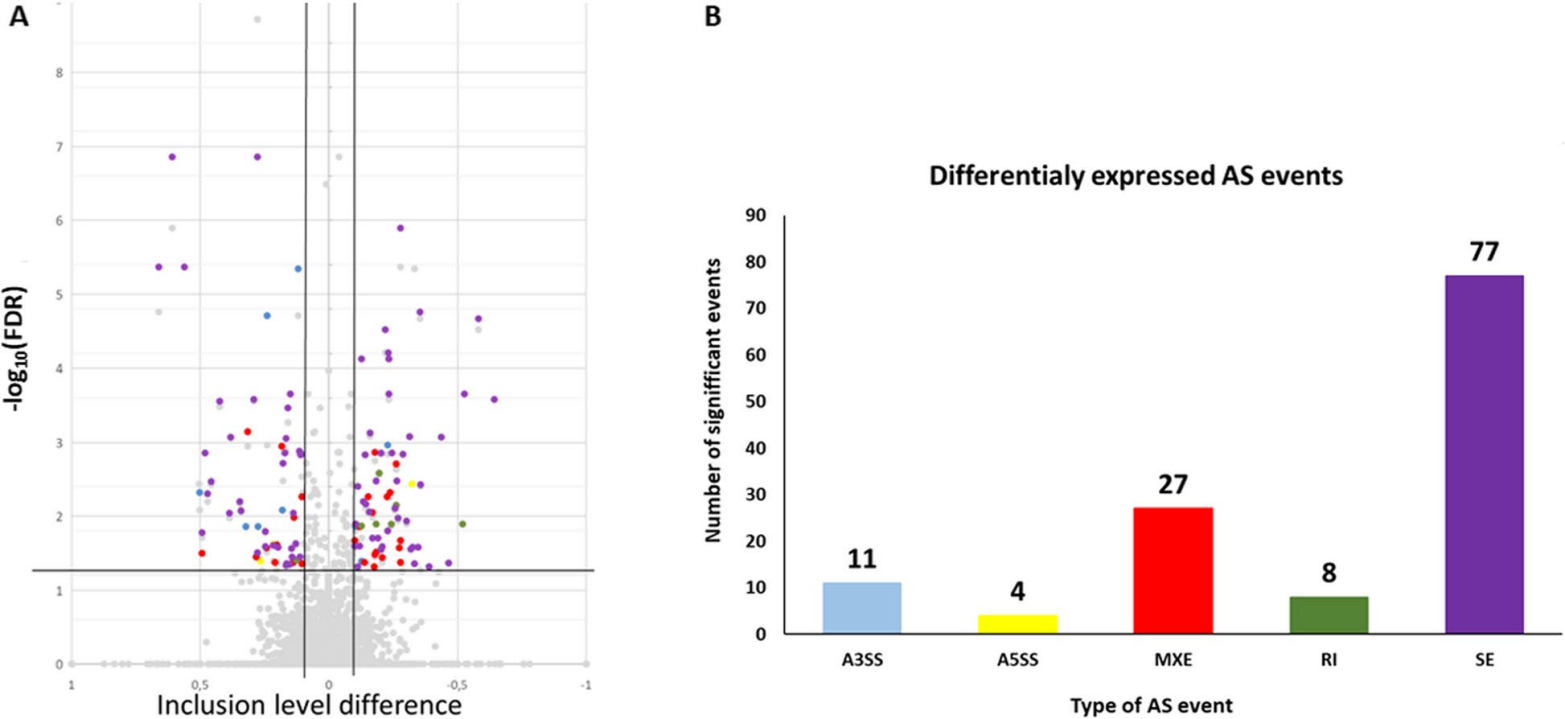
Visualisation of results obtained during alternative splicing (AS) events analysis of AP cells transcriptome under the influence of visfatin. **A** The Volcano plot describes the differences in AS events frequency between the control and visfatin-treated groups. The *X*-axis describes ΔPSI-values for each AS event and the* Y*-axis shows the negative decimal logarithm of the adjusted *P*-value. The horizontal line refers to the negative logarithmic adjusted *P*-value cut-off (q = 1.3). The vertical lines refer to the ΔPSI-value cut-off (ΔPSI > |0.1|). **B** Bar graph presenting the occurrence of differentially expressed AS events (DAS). The vertical axis presents the number of DASs, and the horizontal axis presents different types of alternative splicing events. Different colours of dots (A) and bars (B) refer to the different types of significant AS. Events: blue — alternative 3′ splicing sites (A3SS), yellow — alternative 5′ splicing sites (A5SS), red — mutually exclusive exons (MXE), dark green — retention intron (RI), and purple — skipping exon (SE)

**Fig. 8:**
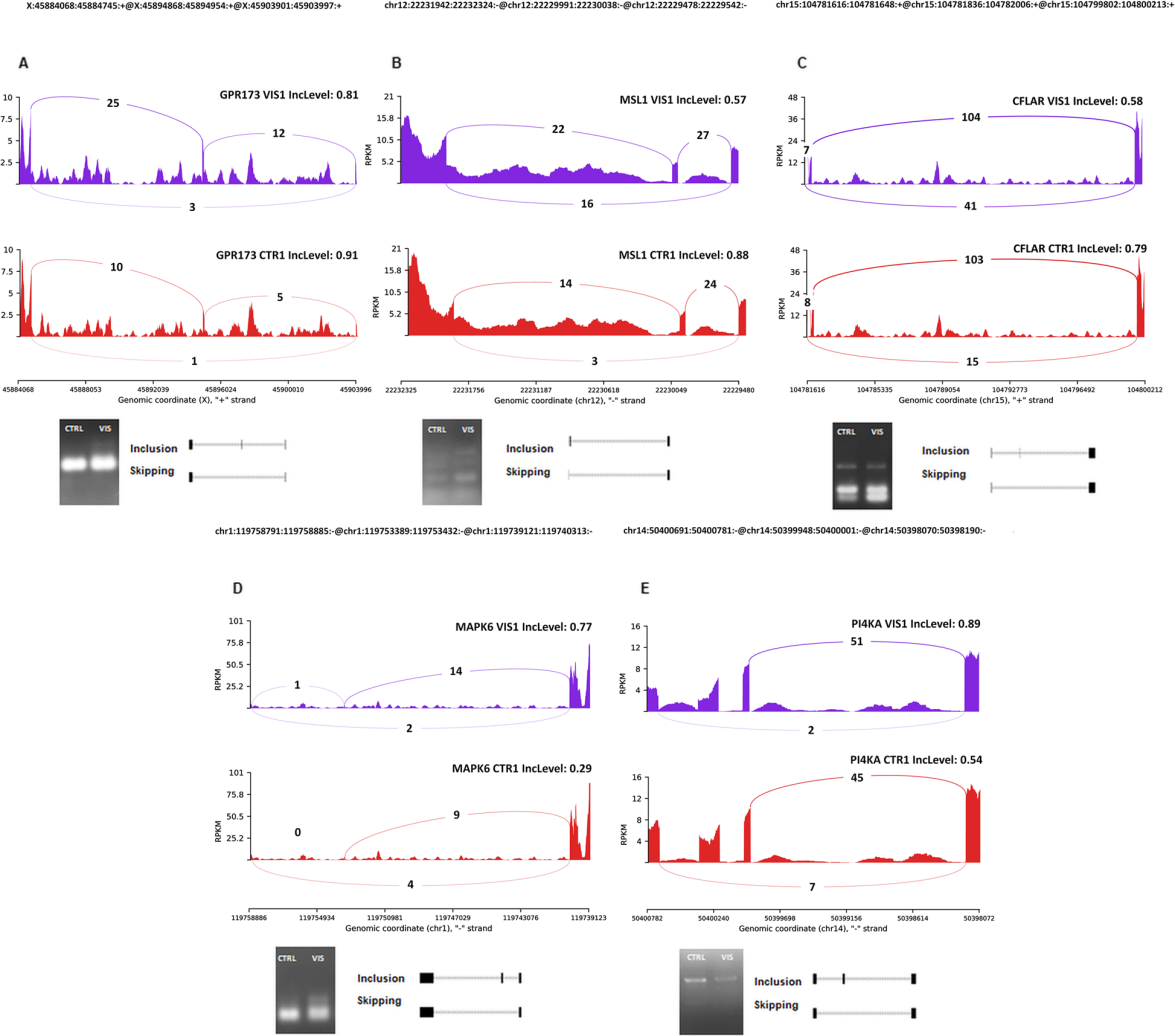
Quantitative visualisation (Sashimi plot) of differential alternative splicing events (DASs) statistically significant in the changes of percentage splicing inclusion (*q* < 0.05 and |ΔPSI| > 0.1) between visfatin-treated group (VIS1; purple) and control group (CTRL; red) samples. The numbers in each upper right corner of the tracks present the percentage of splicing inclusion (PSI) values. Splicing differentiations are covered by the number of reads mapped in the range of junction sites. The upper black tracks show the genomic localisation of splicing events. The plot was generated in ggsashimi Python script. Below of plots, we present validation results of chosen DASs using the PCR method. The images show the inclusion and skipping exon levels between VIS and the control group. Abbreviations: GPR173 - G protein-coupled receptor 173, MSL1 - MSL complex subunit 1, CFLAR - CASP8 and FADD like apoptosis regulator, MAPK6 - mitogen-activated protein kinase 6, PI4KA - phosphatidylinositol 4-kinase alpha

The analysis of AS occurrence revealed 21,736 events, from which we identified 235 as belonging to the DASs group (|ΔPSI| > 0.1; Fig. 7). Among all the revealed events, we identified 11 A3SS, 4 A5SS, 27 MXE, 8 RI, and 77 SE events. Figure 8 presents the Sashimi plots of the chosen DASs (*GPR173, MSL1, CFLAR, MAPK6* and *PI4KA*). The detailed results of the DASs analysis are provided in Supplementary File 5.

### Quantitative real-time PCR and PCR analyses results

**Fig. 9:**
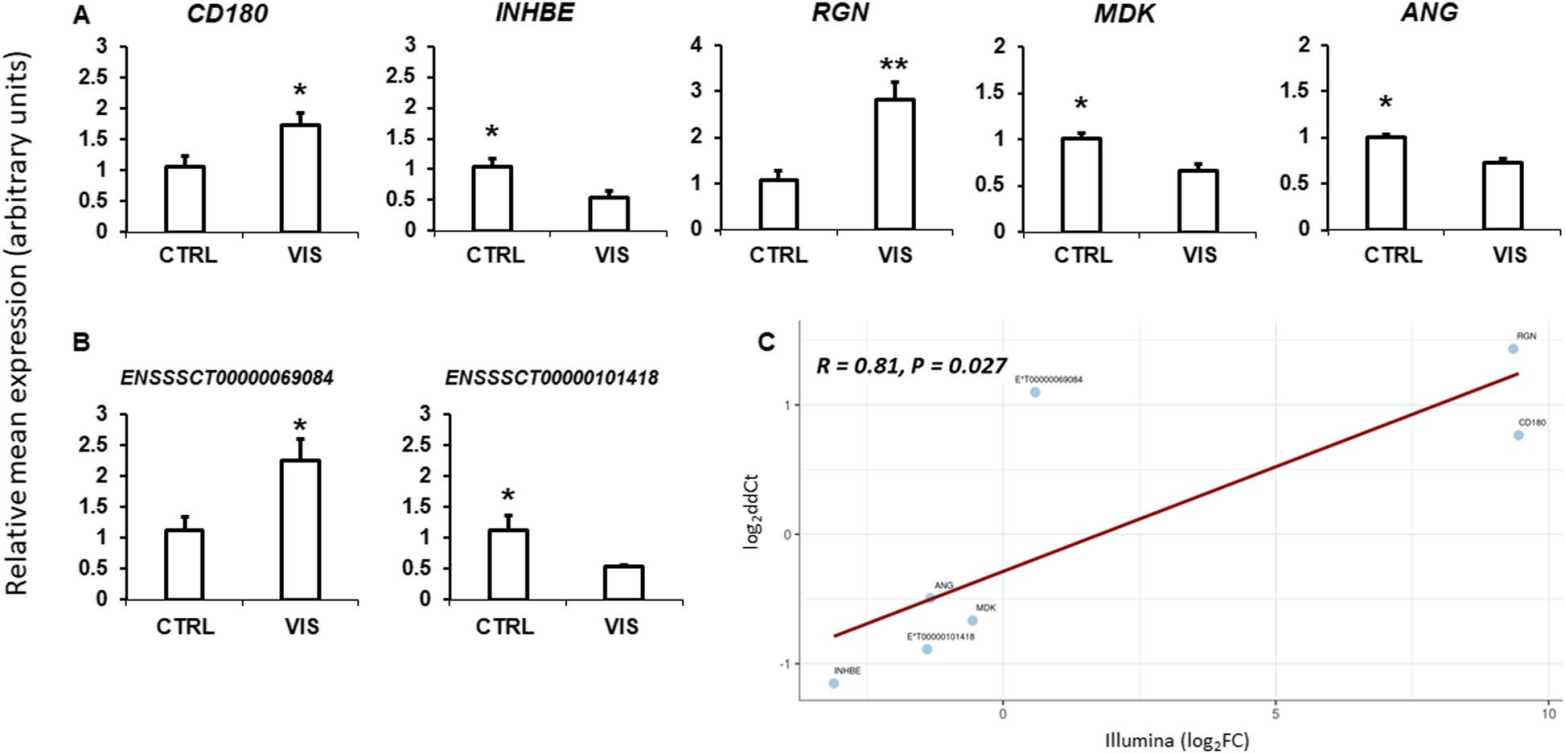
Quantitative real-time PCR validation of RNA-seq results for differentially expressed genes (**A**) and long noncoding RNAs (**B**) in the visfatin-treated porcine anterior pituitary cells. Panel (**C**) presents the correlation between the obtained qPCR and RNAseq results. Validation was performed for *CD180*, *INHBE*, *RGN*, *MDK* and *ANG* genes, as well as for *ENSSSCT00000069084* and *ENSSSCT00000101418* lncRNAs with the use of UBC (ubiquitin C) and 18sRNA (18S ribosomal RNA) reference genes (* *P*-value < 0.05; ** *P*-value < 0.01). Abbreviations: CTRL - control group, VIS - visfatin-treated group, CD180 - CD180 molecule, INHBE - inhibin subunit beta E, RGN - regucalcin, MDK – midkine, ANG - angiogenin

To validate the obtained RNA-seq results, five DEGs (*CD180*, *INHBE*, *RGN*, *MDK* and *ANG*), as well as 2 DELs (*ENSSSCT00000101418* and *ENSSSCT00000069084*) were selected for the qPCR experiment. For the validation of DASs analysis, five AS genes were chosen (*GPR173, MSL1, CFLAR, MAPK6* and *PI4KA*). QPCR analyses of DEGs and DELs (Fig. 9A and B) and PCR analysis of DASs (Fig. 8) confirmed the data obtained during the NGS experiment. Validation results confirmed the veracity and accuracy of the RNA-Seq, as well as the data analysis methods used in the present study.

## Discussion

To our knowledge, this is the first study describing the influence of VIS on the global gene expression in mammalian cells. Until today, the only study describing the influence of the hormone on the transcriptome was conducted with the in vivo use of VIS in chicks [60]. Herein, we revealed the significant effect of the hormone on the expression of several genes and lncRNAs, as well as on the occurrence of DASs in the porcine APc. The created interaction analysis presents a complex network of genes interplaying with each other on different levels, from which the most common was co-expression and physical interactions. Despite the low number of direct interactions between them, their interdependence through the intermediate genes despite completely different functions indicates the complex and pleiotropic influence of VIS in the porcine pituitary.

Visfatin, as one of the adipokines responsible for the regulation of metabolism, is also perceived as a potential factor integrating energy balance and endocrine system regulation in response to the hormonal status of the organism. The expression of the VIS gene and protein has been reported in different endocrine organs such as sheep, mice, porcine and human pituitaries [34, 61, 62], human, bovine, porcine and murine ovaries [32, 62–64], rat, pig, mice, chicken and human testes [33, 65–68]. Besides the direct expression of VIS in the endocrine glands, the hormone expression was also confirmed in mice and porcine hypothalami, the overriding structure responsible for the regulation of the endocrine functions [29, 67]. Therefore, it seems that VIS may play a significant role in the HPG axis’ functioning. Evidence for this may be provided by the research results of Maillard et al. [62], who indicated the inhibitory influence of adipokine on the secretion of LH by the mice LβT2 cells. The hormone was also found to stimulate the secretion of adrenocorticotrophic hormone (ACTH) by rat corticotroph cells [69]. It was also proven that VIS is involved in the regulation of ovarian and testicular steroidogenesis [32, 70, 71].

During the present study, we indicated the influence of VIS on the expression of genes connected with the regulation of pituitary secretory properties. The adipokine stimulated the expression of progesterone receptor (*PGR*) and early growth response 2 (*EGR2*) genes and inhibited the expression of inhibin subunit beta E (*INHBE*), PROP Paired-Like Homeobox 1 (*PROP1*), and dopamine receptor 2 (*DRD2*) genes. Progesterone receptors (PRs) mediate the actions of the progestin hormones influencing reproductive functions. PRs belong to the group of intracellular factors acting as ligand-modulated transcription agents [72]. The enhanced expression of the member of the ‘steroid hormone mediated signalling pathway’ (GO: GO:0043401), *PGR*, in the APc under the influence of VIS indicates its potential involvement in the regulation of the gland sensitivity to P_4_ and, indirectly, on the relationship between the pituitary and ovaries, and ovarian processes, such as steroidogenesis, as indicated by Reverchon et al. [32]. Another member of the mentioned term, retinoid X receptor gamma (*RXRG*) has also a proven role in the regulation of progesterone, as well as oestradiol-mediated transcription processes [73]. Besides its regulatory influence on the APc physiology through *PGR*, VIS affected also several other genes connected with the regulation of females’ reproductive functions. We revealed the inhibition of *INHBE* gene expression under the influence of VIS. Inhibins and activins belong to the group of peptide hormones of the transforming growth factor-β superfamily and play a pivotal role in the regulation of the reproductive system functioning [74], controlling, i.a., FSH release from the pituitary [75]. The direct perfusion of inhibin into the pituitary causes a drastic decrease in follicle-stimulating hormone subunit beta (*FSHβ*) gene expression and, for more, decreases the half-life of *FSHβ* subunit mRNA [76, 77].

These indicates that VIS may be involved in the regulation of gonadotrophins production during the oestrous cycle which seems to be confirmed by the revealed change in the occurrence of skipping exon events in the G protein-coupled receptor 173 (*GPR173*) gene (ΔPSI = 0.103) encoding the phoenixin receptor. Phoenixin is a neuropeptide with a proven regulatory role in gonadotrophins’ secretion through the modulation of gonadoliberin (GnRH) receptor expression [78]. It was hypothesized that phoenixin may regulate the preovulatory LH surge required for ovarian cyclicity [78, 79]. Since Stein et al. [79] reported that silencing of the *GPR173* gene abrogates the phoenixin-induced LH secretion, we may conclude that through the modulation of *GPR173* splicing frequency and *INHBE* expression, VIS affects the production of gonadotrophins, and hence, affects the ovarian functions. The confirmation of the above may be supported by the results of our studies indicating the modulatory effect of VIS on basal and GnRH-stimulated secretion of LH and FSH by the porcine APc [35].

Besides the modulatory influence of VIS on the gonadotroph cells in the AP, its influence on the expression of genes mentioned above indicates its regulatory effect on the secretion of other pituitary-derived hormones such as PRL, growth hormone (GH) and thyroid stimulating hormone (TSH). We revealed the inhibitory effect of adipokine on the expression of the *PROP1* gene, which product is crucial for pituitary development and physiology. The pituitaries of *PROP1* null mice were found to be hypoplastic and the gonadotroph cell differentiation was delayed [80]. Patients with the defect in the *PROP1* gene are characterized by the impaired production of GH, TSH and PRL, as well as LH and FSH [81]. On the other hand, the overexpression of the *PROP1* gene was observed in pituitary adenomas which indicates its crucial role in pituitary development [82]. The product of another VIS-affected gene encodes EGR2, also connected with GH production which inhibition results in the somatotroph cell population ablation [83]. The next revealed gene, *DRD2*, encodes dopamine receptor D2. At the level of the pituitary, dopamine inhibits PRL secretion via the DRD2 receptor [84–86]. VIS influence on the lactotroph cells’ proliferation and secretory functions through modulation of *DRD2* expression may be additionally supported by its effect on the modulation of DASs in the latent transforming growth factor beta binding protein 4 (*LTBP4*) gene (RI; ΔPSI = 0.163). LTBP4 is responsible for enhancing the secretion of the TGF-β latent form, regulating the availability and activation of the growth factor. TGF-β was found to cooperate with dopamine in the regulation of lactotroph functions [87, 88]. The above-presented findings indicating the modulatory influence of VIS on the expression of genes connected with the pituitary development and production of hormones reveal the existence of complex mechanisms of the pituitary trophic cell regulation, in which VIS may play a crucial role.

Besides its primary function as the metabolic regulator, VIS plays an important role in several processes such as apoptosis, cell proliferation, or angiogenesis. VIS, through the mitogen-activated protein kinase/phosphoinositide 3-kinase (MAPK/PI3K) pathway, may inhibit the apoptosis in the pancreatic MIN6 β-cell line [89]. Xiang et al. [90] reported that the suppression of apoptosis in rat pancreatic β-cell lines is mediated through AMPK and ERK1/2 signalling pathways. In contrast, Li et al. [91] identified that in macrophages the anti-apoptotic influence of VIS is achieved through the interleukin-6/STAT3 signalling mechanism. The above suggests that VIS controls cells’ apoptosis through many different transduction pathways and its activity is complex and multi-pronged. The presented data analysis revealed changes in the expression and occurrence of AS events in several genes connected with apoptosis. VIS inhibited the expression of apoptosis-associated tyrosine kinase (*AATK*) and KLF transcription factor 2 (*KLF2*) and enhanced the expression of regucalcin (*RGN*), caspase activity and apoptosis inhibitor 1 (*CAAP1*), and signal transducing adaptor family member 1 (*STAP1*) genes. AATK acts as the inductor of the apoptosis process [92–95]. The overexpression of *AATK* in melanoma cells caused their growth arrest and induced apoptosis, whereas its epigenetic silencing was observed in human and murine cancer cell lines [96–98]. The product of another gene, *KLF2*, a member of Kruppel-like transcriptional factors, is known for its growth inhibitory and apoptosis induction properties and its expression suppression has been reported in many types of cancer [99–101]. Contrary to the previous genes, the products *RGN, CAAP1*, and *STAP1*, have been found to exert an anti-apoptotic effect and the disruption in their expression was reported in many cancers [102–107]. The mentioned earlier suggestion of VIS involvement in the regulation of the apoptosis process may also be confirmed by its influence on lncRNA expression and AS events in apoptosis-related genes. The analyses revealed a significant influence of VIS on lncRNA addressed to GO terms connected to apoptosis-related signalling pathways, ‘positive regulation of intrinsic apoptotic signaling pathway by p53 class mediator’ (GO:1,902,255) and ‘positive regulation of NIK/NF-kappaB signaling’ (GO:1,901,224). We indicated the modulatory effect of the adipokine on the expression of *ENSSSCT00000101418* lncRNA transcript interacting with the cytoskeleton-associated protein 5 (*CKAP5*) gene which is engaged in apoptosis. Cells of different tumour cell lines were reported to be arrested in metaphase and underwent cell death after silencing *CKAP5* [108]. We identified AS modifications of mRNA encoding *MSL1* (SE; ΔPSI= −0.314), *CFLAR* (SE; ΔPSI=−0.208), solute carrier family 25 member 23 (*SLC25A23*; RI; ΔPSI= −0.243), and ILK associated serine/threonine phosphatase (*ILKAP*; RI; ΔPSI = 0.127) genes, whose products are connected with the process of apoptosis [109–113]. The above confirms that VIS is an important factor in the regulation of APc survival through its role in the control of apoptosis.

Another important feature of VIS is the hormone’s influence on the process of angiogenesis. Through the stimulation of vascular endothelial growth factors (VEGF) and matrix metalloproteinases (MMP) production, the adipokine enhances endothelial angiogenesis. Additionally to the previous, VIS was also reported to inhibit the production of MMPs inhibitors, TIMP Metallopeptidase Inhibitors 1 and 2 (TIMP-1 and TIMP-2) [114]. Another study confirms the pro-angiogenic actions of VIS which takes place through the stimulation of interleukin-6 expression in endothelial cells [115]. Herein we revealed the modulatory effect of VIS on the gene connected with ‘positive regulation of vasculogenesis’ (GO:2001214), *CD34*, as well as on the expression of several genes connected with angiogenesis, like the endosialin gene (*CD248), KLF2, MDK*, and *ANG*. VIS caused an increase in the expression of the *CD248* gene which was reported as a potentially important marker of embryonic and tumour vascularization. Since CD248 has not been reported in endothelial cells, it is considered a marker of neighbouring pericytes and stromal cells influencing the process of new vessel formation [116–119]. Contrary to CD248, *KLF2* product has been found to inhibit endothelial cell activation and proliferation, as well as VEGF-A-mediated angiogenesis [120]. The VIS-mediated stimulation of the *CD248* gene and suppression of the *KLF2* gene suggest its pro-angiogenic features. However, VIS also suppressed the expression of other pro-angiogenic factors, *ANG*, *MDK*, and *CD34.* Angiogenin is a crucial factor for the induction of angiogenesis through the activation of cell migration, invasion, proliferation, and formation of tubular structures [121]. In tumorigenesis, ANG, secreted by developing tumour cells, ensures their nutrition, through the stimulation of vessels, as well as the promotion of cells’ proliferation [122–124]. Midkine, besides its role in the promotion of angiogenesis, takes also part in the regulation of cell migration and neurogenesis. The downregulation of MDK results in suppression of angiogenesis, independently of VEGF [125–127]. CD34 is considered a marker of the vascularization process [128]. The ambiguous influence of VIS on the expression of genes connected with angiogenesis suggests that adipokine may play a role in maintaining homeostasis of the angiogenesis process through the regulation of new vessel formation. That thesis may be also supported by the results concerning the alteration in lncRNAs’ expression and AS occurrence. We discovered that the presence of VIS altered the frequency of AS events in the integrin subunit alpha V (*ITGAV*) gene (A3ss; ΔPSI= −0.128). The product of *ITGAV*, integrin αV, which together with integrin β3 forms a vitronectin receptor, has been found to play a key role in the new vessel formation. The suppression of the vitronectin receptor by its antagonists resulted in the inhibition of the vessels’ formation in many experimental models [129–133]. What is more, we identified also VIS-induced changes in the expression of lncRNAs connected with cytoplasmic polyadenylation element binding protein 1 (*CPEB1*) and AKT serine/threonine kinase 3 (*AKT3*) genes (*ENSSSCT00000069084* and MSTRG.1914, respectively). Both genes were found to be involved in angiogenesis [134–137]. The alteration in the expression of their regulatory lncRNAs taken together with the proven influence of VIS on the expression of DEGs and AS events frequency confirms the adipokine’s involvement in the regulation of this process in the glandular part of the porcine pituitary.

## Conclusions

To our knowledge, this is the first study concerning the influence of VIS on the global gene expression in the mammalian APc. The revealed influence of the adipokine on the expression of genes, such as those important for the secretory functions of the gland as *PGR, INHBE*, and *DRD2*, taken together with its influence on genes involved in the apoptosis (*RGN*) and angiogenesis (*ANG*), presents VIS as an important agent in the regulation of pituitary physiology. Taking into consideration that all three mentioned processes are crucial in the process of tumorigenesis, the results presented herein may help to understand this process and the potential role of VIS in pituitary tumour formation, however, further, in-depth studies are necessary.

### Supplementary Information

**Supplementary File** **1**. General statistics of high-throughput RNA sequencing and mapping to the porcine genome.

**Supplementary** **File 2**. List of differentially expressed genes.

**Supplementary** **File** **3**. Gene Ontology (GO) analysis generated from up-regulated and down-regulated DE-genes.

**Supplementary** **File** **4**. Gene Ontology (GO) analysis generated from up-regulated and down-regulated DELs.

**Supplementary  File 5**. Results obtained during the analysis of differentially expressed AS events

**Supplementary** **File** **6**. Complete list of gene interactions generated by GeneMania.

## Data Availability

The datasets supporting the conclusions of this article are available in the Functional Genomics Data Collection (ArrayExpress) repository and can be accessed with the E-MTAB-13922 accession number.

## Abbreviations

18sRNA: 18S ribosomal RNA
A3SS: Alternative 3′ splice site (alternative splicing type)
A5SS: 5′ splice site (alternative splicing type)
AATK: Apoptosis-associated tyrosine kinase
ACTH: Adrenocorticotropic hormone
ANG: Angiogenin
AP: Anterior pituitary
APc: Anterior pituitary cells
AS: Alternative splicing
BP: Biological processes (GO category)
CAAP1: Caspase activity and apoptosis inhibitor 1
CC: Cellular components (GO category)
CD180: CD180 molecule
CD248: Endosialin
CFLAR: CASP8 and FADD-like apoptosis regulator
CKAP5: Cytoskeleton associated protein 5
CPEB1: Cytoplasmic polyadenylation element binding protein 1
DASs: Differential alternative splicing events
DEGs: Differentially expressed genes
DELs: Differentially expressed lncRNAs
DRD2: Dopamine receptor 2
EGR2: Early growth response 2
FC: Fold change
FDR: False discovery rate
FSH: Follicle-stimulating hormone
GH: Growth hormone
GnRH: Gonadotrophin-releasing hormone
GO: Gene Ontology
GPR173: G protein-coupled receptor 173
HPG: Hypothalamic-pituitary-gonadal axis
INHBE: Inhibin subunit beta E
KEGG: Kyoto Encyclopedia of Genes and Genomes
KLF2: KLF transcription factor 2
LH: Luteinizing hormone
lncRNAs: Long noncoding RNAs
MAPK6: Mitogen-activated protein kinase 6
MDK: Midkine
MF: Molecular function (GO category)
MMP: Matrix metalloproteinases
MSL1: MSL complex subunit 1
MXE: Mutually exclusive exons (alternative splicing type)
NAMPT: Nicotinamide phosphoribosyltransferase
NGS: Next generation sequencing
P_4_: Progesterone
PGR: Progesterone receptor
PI4KA: Phosphatidylinositol 4-kinase alpha
PRL: Prolactin
PROP1: PROP Paired-Like Homeobox 1
PRs: Progesterone receptors
qPCR: Quantitative real-time polymerase chain reaction
RGN: Regucalcin
RI: Retention intron (alternative splicing type)
RT-PCR: Reverse transcription polymerase chain reaction
SE: Skipping exon (alternative splicing type)
TSH: Thyroid stimulating hormone
UBC: Ubiquitin C
VEGF: Vascular endothelial growth factors
VIS: Visfatin
